# Interplay between mistranslation and oxidative stress in *Escherichia coli*

**DOI:** 10.2478/aiht-2024-75-3834

**Published:** 2024-06-29

**Authors:** Valentina Ević, Jasmina Rokov-Plavec

**Affiliations:** University of Zagreb Faculty of Science, Department of Chemistry, Zagreb, Croatia

**Keywords:** aminoacyl-tRNA synthetase, hydrogen peroxide, isoleucyl-tRNA synthetase, stress response, translation, aminoacil-tRNA-sintetaza, izoleucil-tRNA-sintetaza, odgovor na stres, translacija, vodikov peroksid

## Abstract

Mistakes in translation are mostly associated with toxic effects in the cell due to the production of functionally aberrant and misfolded proteins. However, under certain circumstances mistranslation can have beneficial effects and enable cells to preadapt to other stress conditions. Mistranslation may be caused by mistakes made by aminoacyl-tRNA synthetases, essential enzymes that link amino acids to cognate tRNAs. There is an *Escherichia coli* strain expressing isoleucyl-tRNA synthetase mutant variant with inactivated editing domain which produces mistranslated proteomes where valine (Val) and norvaline (Nva) are misincorporated into proteins instead of isoleucine. We compared this strain with the wild-type to determine the effects of such mistranslation on bacterial growth in oxidative stress conditions. When the cells were pre-incubated with 0.75 mmol/L Nva or 1.5 mmol/L Val or Nva and exposed to hydrogen peroxide, no beneficial effect of mistranslation was observed. However, when the editing-deficient strain was cultivated in medium supplemented with 0.75 mmol/L Val up to the early or mid-exponential phase of growth and then exposed to oxidative stress, it slightly outgrew the wild-type grown in the same conditions. Our results therefore show a modest adaptive effect of isoleucine mistranslation on bacterial growth in oxidative stress, but only in specific conditions. This points to a delicate balance between deleterious and beneficial effects of mistranslation.

## Sažetak

Pogreške u translaciji uglavnom su povezane s toksičnim učincima u stanici zbog proizvodnje funkcionalno aberantnih i pogrešno smotanih proteina. Međutim, pod određenim okolnostima pogreške u translaciji mogu imati povoljne učinke i omogućiti stanicama da se prilagode drugim stresnim uvjetima. Mistranslacija može biti prouzročena pogreškama koje čine aminoacil-tRNA-sintetaze, esencijalni enzimi koji povezuju aminokiseline s pripadnim molekulama tRNA. Soj bakterije *Escherichia coli* koji eksprimira mutiranu inačicu izoleucil-tRNA-sintetaze s inaktiviranom domenom za popravak, proizvodi mistranslatirane proteome u kojima su valin (Val) i norvalin (Nva) pogrešno ugrađeni u proteine umjesto izoleucina. Usporedili smo ovaj soj s divljim tipom kako bismo odredili učinke takve mistranslacije na rast bakterija u uvjetima oksidacijskog stresa. Kada su stanice bile prethodno inkubirane s 0,75 mmol/L Nva ili 1,5 mmol/L Val ili Nva i potom izložene vodikovu peroksidu, nije primijećen povoljan učinak mistranslacije. Međutim, kada je soj s nedostatkom popravka pogreške uzgajan u mediju s dodatkom 0,75 mmol/L Val do rane ili srednje eksponencijalne faze rasta te potom izložen oksidacijskom stresu, prerastao je divlji tip uzgojen u istim uvjetima. Stoga naši rezultati pokazuju skroman adaptivni učinak mistranslacije izoleucina na rast bakterija u oksidacijskom stresu, ali samo u određenim uvjetima. To upućuje na osjetljivu ravnotežu između štetnih i povoljnih učinaka mistranslacije.

Translation is a fundamental cellular process in which the nucleotide sequence of mRNA is translated into the amino acid sequence of a protein. The important role in this process is played by aminoacyl-tRNA synthetases (aaRSs), enzymes which covalently link amino acids to cognate tRNAs. The resulting aminoacyl-tRNAs (aa-tRNA) serve as substrates during translation on the ribosome (1, 2). Additionally, many aaRSs are multifunctional proteins participating in various cellular processes beyond translation, including stress response (3, 4, 5, 6, 7, 8).

Aminoacylation is a two-step reaction. The first, activation step, involves adenosine triphosphate (ATP) to form an aminoacyladenylate (aa-AMP) intermediate. The second step involves the transfer of the aminoacyl moiety to cognate tRNA. Due to the structural and chemical similarity of some amino acids, certain aaRSs can mistake non-cognate for cognate amino acids, which can lead to their incorporation in the protein and result in mistranslation (mistake in translation). To reduce the rate of mistranslation, aaRSs correct their mistakes through editing reactions, which can occur before or after the transfer of the aminoacyl group to the tRNA (9, 10). Pre-transfer editing involves the hydrolysis of non-cognate aa-AMP in the synthetic site (where aminoacylation takes place), while post-transfer editing involves the hydrolysis of non-cognate aa-tRNA in a specialised editing domain.

Isoleucyl-tRNA synthetase (IleRS) charges tRNA^Ile^ with cognate amino acid isoleucine (Ile) enabling its incorporation in the proteins. IleRS has been known to mistake proteinogenic amino acid valine (Val) and non-proteinogenic amino acid norvaline (Nva) for Ile (11, 12). Nva is a non-canonical amino acid and a side-product of leucine biosynthesis in bacteria (13). To repair the result of non-cognate amino acid misrecognition IleRS uses both pre-transfer editing of non-cognate aa-AMP within the synthetic site and deacylation of misaminoacylated tRNA^Ile^ in the post-transfer editing domain (11, 12, 14, 15).

Mistranslation is usually toxic as it leads to the production of functionally aberrant and misfolded proteins, which is often associated with adverse effects due to protein aggregation in the cell, impaired cell fitness and growth, morphological changes, and even cell death (16, 17, 18). In yeast, mistranslation disrupts the mitochondrial function (19), in a mouse model misfolded proteins cause neurodegenerative diseases (20), while mutations increasing the rate of mistranslation are linked to neuropathies in humans (21, 22). Aminoglycoside antibiotics, such as streptomycin and kanamycin, kill bacteria because they induce mistranslation and protein misfolding (23). In *Escherichia coli* mistranslation at isoleucine positions causes proteotoxic stress which provokes the SOS response and global proteome dysregulation and upregulates cellular apparatus to maintain proteostasis by increasing the levels of major chaperones, proteases, and disaggregase ClpB (24).

However, there are several examples in which mistranslation has a beneficial effect (25, 26). Naturally occurring mistranslation of specific amino acids can be induced as part of response to stress. A most notable example is mismethionylation induced under oxidative stress, which is found in all three domains of life (25, 27). It stems from the ability of methionine to act as a sink for reactive oxygen species (ROS), since it can be reversibly oxidised to methionine sulphoxide (28). Constitutively higher mistranslation rates are common in parasites, such as *Mycoplasma* (29, 30) and mycobacterial pathogens (31), which are associated with antibiotic resistance and may lead to antigen diversity, enabling pathogens to escape the host's immune system. In some cases, mistranslation induces general stress response, resulting in tolerance to other stress conditions (25, 26).

Reactive oxygen species, such as superoxide, hydrogen peroxide, and hydroxyl radicals, are by-products of aerobic metabolism and play an important role in cell signalling in many organisms, including bacteria (32, 33). However, if the metabolism is disrupted, ROS levels increase beyond the cell's capacity to clear them, which causes oxidative stress (34). Additionally, oxidative stress can be caused by other bacteria that produce and excrete ROS or by plant cells and animal immune cells that produce ROS in defence against pathogenic bacteria (35). Furthermore, oxidative stress may be a result of microbe exposure to heat, metals, solvents, and clinical antibiotics (35). Whichever the cause, oxidative stress damages nucleic acids, lipids, and proteins and, ultimately, kills the cell (34). It also has diverse effects on the aaRS function. In most cases it impairs the aaRS activity (36, 37, 38, 39), but some aaRSs are resistant to oxidative conditions (40, 41, 42, 43).

We know little about how mistranslation affects response to oxidative stress in the *Escherichia coli* strain expressing IleRS mutant variant with inactivated editing domain, which produces mistranslated proteomes by mistaking valine and norvaline for isoleucine (12, 44). Therefore, we compared this strain with the wild-type to decipher the effect of mistranslation of isoleucine with valine or norvaline on the bacterial growth in oxidative stress conditions.

## MATERIALS AND METHODS

### Bacterial strains and growth media

The wild-type *E. coli* strain MG1655 was obtained from the Coli Genetic Stock Center (Yale University, New Haven, CT, USA). To compare the effects of mistranslation we used the MG1655 strain with editing-deficient IleRS [IleRS(Ala_10_) ED^─^, PS7066]. In this strain the wild-type chromosomal IleRS gene is replaced with a mutant IleRS gene in which the coding sequence for conserved threonine-rich peptide (T241–N250) in the editing domain is substituted with the coding sequence for 10 consecutive Ala residues (44). The resulting protein IleRS(Ala_10_) does not have a functional editing domain and can not hydrolyse misaminoacylated tRNAs^Ile^, Val-tRNA^Ile^, and Nva-tRNA^Ile^ (12, 44).

Both strains were grown at 37 °C in the M9 minimal medium (BD Biosciences, Franklin Lakes, NJ, USA) supplemented with 2 mmol/L MgSO_2_, 0.1 mmol/L CaCl_2_, 0.4 % glucose, 50 μmol/L thiamine, 100 μmol/L Ile, 100 μmol/L Leu, and 100 μmol/L Val (hereinafter: enriched M9 medium). Considering that higher concentrations of Val inhibit isoleucine biosynthesis, which causes isoleucine pseudo-auxotrophy in strains derived from *E. coli* K12 (13), we added low concentrations (100 μmol/L) of branched-chain amino acids to the medium.

### Determination of growth curves

To compare the growth of the wild-type and the mutant strain with the editing-deficient IleRS in mistranslating conditions, i.e. in the presence of Val or Nva, overnight cultures grown in the enriched M9 medium were diluted to OD_600_=0.04 and grown for another 10 h in the enriched M9 medium either supplemented with 0.75 or 1.5 mmol/L Val or Nva or without the amino acid supplements.

To compare the growth of each strain under oxidative stress, overnight cultures were diluted to OD_600_=0.04 in the enriched M9 medium supplemented with various concentrations of H_2_O_2_ (0, 0.25, 0.5, 0.75, or 1 mmol/L) and grown further for 10 h.

To compare the effects of both mistranslation and oxidative stress overnight cultures of both strains were diluted to OD_600_=0.04 in the enriched M9 medium either supplemented with 0.75 or 1.5 mmol/L Val or Nva or without the amino acid supplements. Diluted cultures were divided in two tubes and further cultured for 10 h. Culture in one tube continued to grow in the same medium, while H_2_O_2_ was added to a final concentration of 0.5 mmol/L to the other tube to induce oxidative stress.

To compare the effect of pre-incubation with Val and Nva before the addition of H_2_O_2_, overnight cultures of both strains were diluted to OD_600_=0.04 with or without Val or Nva (0.75 mmol/L or 1.5 mmol/L) to induce mistranslation and were incubated to reach the early exponential phase (OD_600_=0.2) or mid-exponential phase of growth (OD_600_=0.5). At that point, each culture was divided in two samples and 0.5 mmol/L of H_2_O_2_ was added to one to induce oxidative stress. The growth of bacteria was further monitored for up to 10 h since the initial dilution of overnight cultures.

In all experiments growth curves were determined by measuring OD_600_ over 10 h with the Ultrospec 10 cell density meter (Amersham Biosciences, Piscataway, NJ, USA). Due to the limitations of the instrument, OD_600_ values above 2.0 could not be measured. All experiments were repeated at least two times.

## RESULTS

### Val and Nva effects on the growth of the wild-type and editing-deficient *E. coli* strains

**Figure 1:**
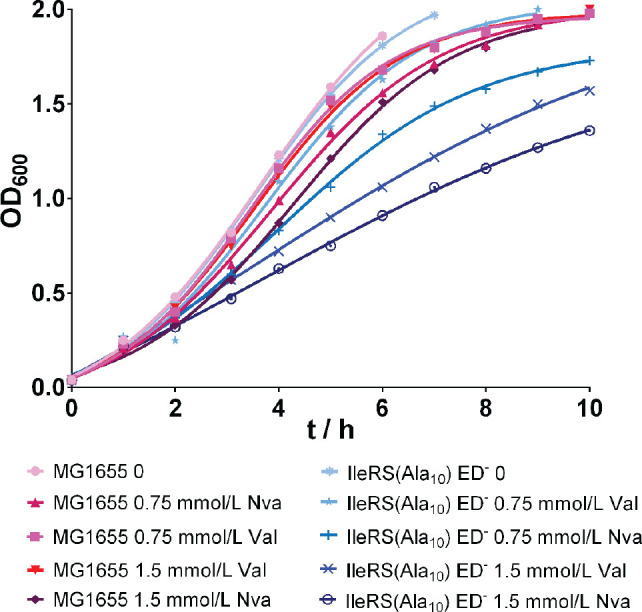
Representative growth curves of at least two biological replicates of the wild-type *E. coli* MG1655 strain and the editing-deficient strain IleRS(Ala_10_) ED^─^ with or without added 0.75 or 1.5 mmol/L valine (Val) or norvaline (Nva)

Figure 1 shows the 10-hour growth curves of both the wild-type and editing-deficient mutant IleRS(Ala_10_) ED^─^ in the enriched M9 medium supplemented with 0.75 or 1.5 mmol/L Val or Nva or not (controls). Val and Nva had a small inhibitory effect on the growth of the wild-type strain, while the inhibition was more pronounced in the editing-deficient strain incubated with both Nva concentrations or 1.5 mmol/L Val. This supports an earlier report that the absence of IleRS editing activity affects the growth due to elevated mistranslation rates when exogenous non-cognate amino acids are present (12). Interestingly, in the presence of 0.75 mmol/L Val the IleRS(Ala_10_) ED^─^ strain growth curve was very similar to the growth curves of the wild-type strain in the presence of both Val concentrations.

### Oxidative stress effects on the growth of the wild-type and editing-deficient *E. coli* strains

**Figure 2:**
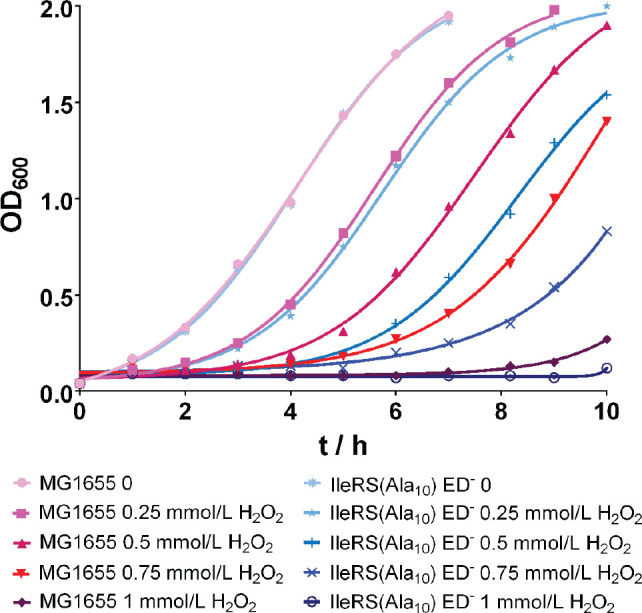
Representative growth curves of at least two biological replicates of the wild-type *E. coli* MG1655 strain and the editing-deficient strain IleRS(Ala_10_) ED^─^ under oxidative stress induced by different concentrations of H_2_O_2_

Figure 2 shows that both the wild-type and the mutant strain were sensitive to H_2_O_2_, which inhibited the bacterial growth by prolonging the lag phase. During the lag phase, bacteria activate stress responses to defend themselves, after which they continue to grow (45, 46, 47, 48). At all H_2_O_2_ concentrations, the mutant exhibited a longer lag phase than the wild-type, which indicates that it took longer to deal with the oxidative stress.

### Growth of the wild-type and editing-deficient *E. coli* strains under simultaneously induced mistranslation and oxidative stress

**Figure 3:**
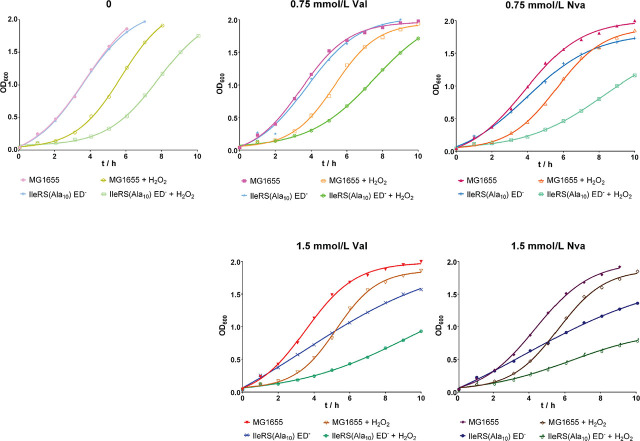
Representative growth curves of at least two biological replicates of the wild-type *E. coli* MG1655 strain and editing-deficient strain IleRS(Ala_10_) ED^─^ in the mistranslating conditions with or without the addition of H_2_O_2_ (final concentration 0.5 mmol/L) to diluted overnight cultures (OD_600_=0.04)

To determine the effects of elevated mistranslation rates induced by Val and Nva on the growth of the editing-deficient mutant strain under oxidative stress, we added 0.5 mmol/L H_2_O_2_ to the cultures, as this concentration had inhibited the growth of both strains but not as much as 0.75 and 1 mmol/L H_2_O_2_. Cell growth was monitored for 10 h (Figure 3). Unsupplemented with Val, Nva, or H_2_O_2_, both strains grew similarly, but supplementation with 0.5 mmol/L H_2_O_2_ prolonged the lag phase of both strains, albeit more evidently of the editing-deficient strain (Figure 3, top left panel).

In the presence of 0.75 mmol/L Val and H_2_O_2_, the wild-type strain exhibited a longer lag phase compared to conditions without oxidative stress (Figure 3, top middle panel). Interestingly, however, after 10 h, the OD_600_ restored to the one measured in the wild-type culture supplemented with 0.75 mmol/L Val but not H_2_O_2_, which suggests successful recovery of the wild-type strain from oxidative stress. The editing-deficient mutant, in turn, showed slower growth in the presence of 0.75 mmol/L Val and H_2_O_2_, indicating that its growth is adversely affected by mistranslation in the oxidising conditions and does not recover like the wild-type strain.

Experiments with 0.75 mmol/L Nva yielded similar results (Figure 3, top right panel). Supplementation with higher Val and Nva concentrations did not improve the growth of the editing-deficient strain under oxidative stress compared to the culture unexposed to H_2_O_2_ or the wild-type culture under oxidative stress (Figure 3, bottom panels). It appears that the editing-deficient strain is more sensitive to H_2_O_2_ than the wild-type strain and that supplementation with Val or Nva and H_2_O_2_ additionally inhibits its growth.

### Growth of the wild-type and editing-deficient strains pre-incubated with Val or Nva before exposure to H_2_O_2_

**Figure 4:**
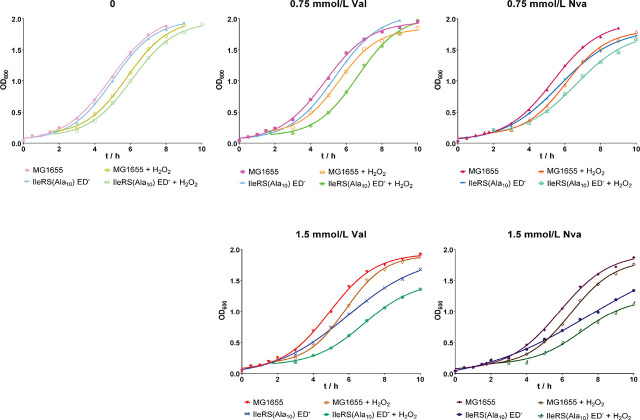
Representative growth curves of at least two biological replicates of the wild-type *E. coli* MG1655 strain and the editing-deficient strain IleRS(Ala_10_) ED^─^ preincubated with Val or Nva with or without the addition of H_2_O_2_ (final concentration 0.5 mmol/L) in the early exponential growth phase (OD_600_=0.2)

Figure 4 shows that H_2_O_2_ added at the early exponential phase (OD_600_=0.2) delayed the growth of both strains when they were not pre-incubated with Val or Nva. However, the lag phase of both strains was shorter (Figure 4, top left panel) than when H_2_O_2_ was added immediately to the culture at OD_600_=0.04 (Figure 3, top left panel), indicating that the bacteria become more resilient to oxidative stress as the culture grows. When 0.75 mmol/L Val was present in the medium, H_2_O_2_ addition at the early exponential phase prolonged the lag phase of both strains (Figure 4, top middle panel). Comparison of OD_600_ at hour 10 showed that the editing-deficient strain slightly outgrew the wild type in oxidative conditions (Figure 4, top middle panel), indicating that pre-incubation with 0.75 mmol/L Val had a certain beneficial effect on the editing-deficient strain under oxidative stress, unlike in the experiment when Val and H_2_O_2_ were added simultaneously (Figure 3, top middle panel). Pre-incubation with Nva or 1.5 mmol/L Val, however, did not achieve a similar beneficial effect (Figure 4, top right, bottom left, and bottom right panels). One of the reasons may be that the cellular response to mistranslation stress did not have enough time to adapt cells to oxidative stress.

**Figure 5:**
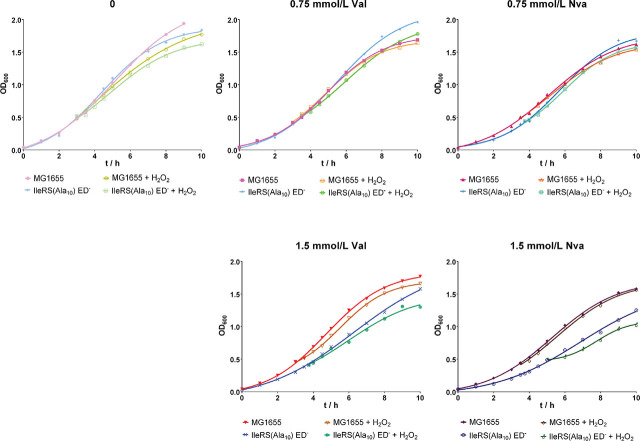
Representative growth curves of at least two biological replicates of the wild-type *E. coli* MG1655 strain and the editing-deficient strain IleRS(Ala_10_) ED^─^ preincubated with Val or Nva with or without the addition of H_2_O_2_ (final concentration 0.5 mmol/L) in the mid-exponential growth phase (OD_600_=0.5)

Therefore, we conducted another experiment in which H_2_O_2_ was added when bacterial cultures reached the mid-exponential phase of growth (OD_600_=0.5). The resulting inhibition was small in both strains (Figure 5, top left panel). In the wild-type strain pre-incubated with 0.75 mmol/L Val the effect of H_2_O_2_ was minimal (Figure 5, top middle panel). As in the previous experiment, the editing-deficient strain eventually outgrew the wild-type under oxidative conditions (Figure 5, top middle panel) at hour 10. Interestingly, pre-incubation with 0.75 mmol/L Nva resulted in similar OD_600_ at hour 10 as the wild-type's (Figure 5, top right panel), which suggests that longer pre-incubation with non-cognate amino acids before the addition of H_2_O_2_ may improve response to oxidative stress through an adaptive effect of mistranslation. However, higher Val or Nva concentration (1.5 mmol/L) did not result in such an effective response (Figure 5, bottom left and right panels), which points to a delicate interplay between mistranslation and oxidative stress and even more delicate balance between deleterious and beneficial effects of mistranslation.

## DISCUSSION

To the best of our knowledge, there are no reports on organisms with editing-deficient aaRSs and their response to oxidative stress. Understanding the impact of mistranslation on the bacterial cell and its response to oxidative stress is important in biomedicine, because the immune system has a toxic effect on pathogenic bacteria via ROS (35). Therefore it is essential to elucidate the mechanisms of adaptation of bacteria to oxidative stress.

In general, mistranslation has toxic effect on cells and organisms (16, 17, 18), but some translational errors can be beneficial for a cell or organism under certain stress conditions (26). For example, global mistranslation of any amino acid caused by ribosomal errors leads to an increased expression of the sigma factor RpoS, a subunit of bacterial RNA-polymerase that directs RNA-polymerase to the promoters of genes associated with stress response (49). Among these genes are the genes coding for catalase KatE and peroxiredoxin OsmC, which are important for the recovery of cells exposed to hydrogen peroxide.

In this work we examined the effects of specific mistranslation at isoleucine codons and replacement of isoleucine with structurally similar Val or Nva on bacterial growth in oxidative conditions. To this end we used the *E. coli* strain expressing IleRS mutant variant with inactivated editing domain, which shows elevated levels of mistranslation especially when the medium is supplemented with Val or Nva (12). When H_2_O_2_ and amino acids were added to overnight cultures, the IleRS(Ala_10_) ED^─^ strain grew more slowly than the wild-type strain, indicating that simultaneous exposure to two types of stress (mistranslation and oxidative stress) is toxic for cells. We have also established that pre-incubation with Nva or 1.5 mmol/L Val before the addition of H_2_O_2_ produced no beneficial effect of mistranslation against oxidative stress. Similar was reported by Pranjic et al. (24), as their high-level isoleucine mistranslation in another IleRS editing-deficient strain lowered bacterial resilience and survival at elevated temperatures. However, when the editing-deficient strain IleRS(Ala_10_) ED^─^ in our study was pre-incubated with 0.75 mmol/L Val until it reached the early or mid-exponential growth and then was exposed to oxidative stress, it slightly outgrew the wild-type cultivated under the same conditions. It appears that the cellular mechanisms of response to mistranslation stress were activated to such an extent that the cells were better prepared to counter oxidative stress. Our findings therefore suggest that there is a delicate balance between deleterious and beneficial effects of mistranslation. It would be interesting to identify cellular mechanisms that allow better growth under oxidative stress due to misincorporation of Val at isoleucine positions in proteins. Additionally, it is possible that other concentrations of Val and Nva as well as different pre-incubation periods would be more beneficial for *E. coli*, which requires further research.

## CONCLUSION

Ours is the first study of how mistranslation caused by IleRS errors affects bacterial growth under oxidative stress. The results show that simultaneous exposure of bacterial cells to two types of stresses (mistranslation and oxidative stress) is toxic and slows down their growth. However, if the cells are first exposed to mistranslation and then oxidative stress (induced in early or mid-exponential growth phase), the toxic effects of oxidative stress are less severe. Interestingly, pre-incubation with the lower Val concentration (0.75 mmol/L) showed even better, yet modest adaptive effect. However, more detailed research is needed to pinpoint conditions in which mistranslation of isoleucine codons confers beneficial effect under oxidative stress and to elucidate molecular mechanisms that enable this adaptation.
